# Anonymity in Kidney Paired Donation: A Systematic Review of Reasons

**DOI:** 10.3389/ti.2023.10913

**Published:** 2023-02-02

**Authors:** Kailing Marcus, Delphine Berner, Karine Hadaya, Samia Hurst

**Affiliations:** ^1^ Institute for Ethics, History, and the Humanities, Faculty of Medicine, University of Geneva, Geneva, Switzerland; ^2^ Service of Nephrology and Hypertension, Geneva University Hospitals and Clinique des Grangettes-Hirslanden, Geneva, Switzerland

**Keywords:** ethics, anonymity, organ transplantation, systematic review, kidney paired donation

## Abstract

The objective of this study was to investigate reasons for or against anonymity that are pertinent to kidney paired donations (KPD). We conducted a systematic review of reasons using PubMed and Google Scholar until May 2022 and through snowballing. Inclusion criteria were publications that: 1) discussed organ donation anonymity; 2) was peer-reviewed; 3) presented at least one reason on anonymity. Exclusion criteria: 1) not published in a scientific journal; 2) grey literature and dissertations. Four researchers independently reviewed and selected papers based on the criteria, extracted text passages and coded them into narrow and broad reason types, selected reasons that were valid for kidney paired donations. 50 articles were included, 62 narrow reasons (*n* = 24 for; *n* = 38 against) and 13 broad reasons were coded. Broad reasons were: protection against harm, general benefits, gratitude, curiosity, unrealistic to implement, fundamental rights, respect people’s wishes, professional neutrality, timing is important, information disclosure, altruism, reciprocity and donation pool. We did not find reasons that justify legal prohibition of donor-recipient interactions for KPD, if they consented to meet. Professional counselling, follow-up and careful evaluations to prevent potential harm.

## Graphical Abstract

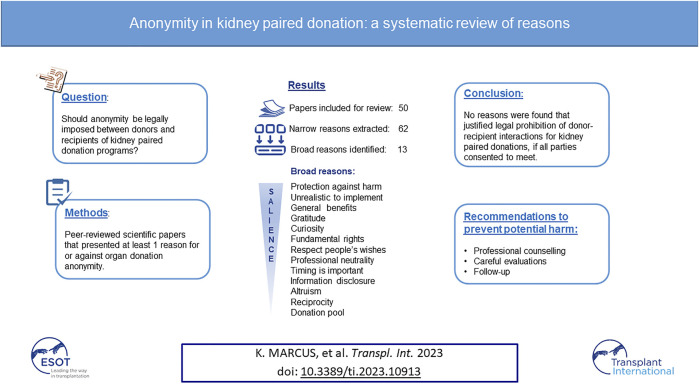

## Introduction

For decades, anonymity has been a core principle in ethical practice of organ donations. The World Health Organization recommends that “personal anonymity and privacy of donors and recipients are always protected” (Guiding Principle 11), and Council of Europe states “anonymity of the donor and of the recipient must be respected” (art. 2.2). Given the intricacies of potential donor-recipient interactions, however, anonymity regulations vary between nations. For instance, Swiss laws on anonymity for paired donation is maintained until pre-surgery, with the possibility of revoking it afterwards, should all concerned persons consent to do so (RS.810.212.3, art. 18). In contrast, anonymity is legally mandated before and after the surgery in European countries, such as Netherlands (1), Spain (Ley 30/1979, art. 4.d) and Sweden (2).

Anonymity legislations are generally applicable to all organ transplant contexts, including unspecified, otherwise known as “non-directed,” “altruistic” or “Samaritan” organ donations, and deceased organ donations. For both types, donors and recipients are unrelated and unknown to each other. In specified donations, also known as “directed” organ donations (the organ is intended for a specified recipient), generally a kidney, the donor-recipient relationship can be of genetic or affective nature, such as associations by partnership, friendship or marriage. For specified donations, when a donor is immunologically incompatible to the intended recipient, kidney paired donation (KPD) programs allow donors to give a kidney in exchange of a compatible one from another donor to their intended recipient. Paired organ donations are thus considered a variant of direct organ donation.

While donor-recipient anonymity is defined by national policies, it is still a subject of debate. The Italian Committee for Bioethics, for instance, petitioned to allow deceased donor families and recipients to make contact, if given explicit consent (3). For KPD, other circumstances further complicate the subject. First, because this donation type involves at least two donation pairs, an individual’s choice to meet the other pair may lead to undesired relationships for the partner involved. Second, in contrast to deceased donor or unspecified donations, a KPD donor’s intent is not entirely altruistic, since both donation pairs have a gain from participating the exchange.

Further, anonymity between organ donation pairs is arguably a question that requires considerations on the reasoning of ethical concerns, to help the policy decision-making process (4). While policy discussions on anonymity persist, reason-based literature on the issue remain scarce.

Therefore, the objective of this study is to investigate the reasonings of whether anonymity should be legally imposed between donors and recipients of KPDs. To do so, we conducted a systematic review of reasons, by investigating reasons presented in peer-reviewed papers for organ donations. We determined those that may be applicable in KPD context, to recommend whether anonymity should be legally imposed, or that it may be relinquished based on free decisions by the donation pairs.

## Materials and Methods

This systematic review of reasons was conducted based on the model by Strech and Sofaer, a method developed for studies that aim to improve argument-based bioethic concerns and to identify gaps that calls for further research (4, 5).

We searched scientific journals in PubMed and Google Scholar databases until May 2022. First, we scanned the databases to identify the appropriate index terms. The search strategy was deliberately wide to broaden the capture of publications, which included editorials, opinion pieces and papers on anonymity for organ donations. We used the string of key terms: (“anonymity” OR “anonymous” OR “confidentiality”) AND (“transplantation” OR “organ donation”) AND (“kidney” OR “renal” OR “liver” OR “hepatic”). Snowballing technique was also applied.

### Inclusion and Exclusion Criteria

A publication was only included if it: 1) presented discussion on donor-recipient anonymity; 2) was peer-reviewed; 3) presented at least one reason for or against anonymity. Papers that were not published in a scientific journal, dissertations, non-peer reviewed publications and grey literature were excluded. No language restrictions were placed, we used DeepL Translator for non-English publications. KM, DB and SH independently reviewed titles and abstract, papers were only included if they met the inclusion criteria. Papers were excluded if at last two reviewers agreed to do so, discrepancies were resolved through discussions.

### Data Synthesis

KM and DB carried out full text analysis to extract text passages that described a reason for or against anonymity, then coded them into “narrow” reasons based on their context. For example, “good relationships were formed” was assigned to passages that described the context of a positive relationship from donor-recipient interactions.

Each “narrow” reason was then coded into “broad” reasons, which gives an overview of the type of reasons in few words. For instance, narrow reasons “donors and recipients are naturally curious about each other” and “direct contact satisfied donor families’ curiosity” were coded as “curiosity” broad reason. For complex text passages that could be assigned more than one “broad” or “narrow” reason types were reassessed based only on the paper’s context to minimize bias. The coded reason types were reviewed by KM, SH and KH for validity, then identified those that were applicable for KPD.

Publications were classified by type and country, based on where the research was conducted or where the donation program took place. For editorials, opinion pieces and essays, the country was determined by the authors’ affiliation. Opinion pieces and essays were coded as “discussion paper”; reports on organ donation programs under “program report”; research studies, editorials and conference reports were coded accordingly.

## Results

**FIGURE 1:**
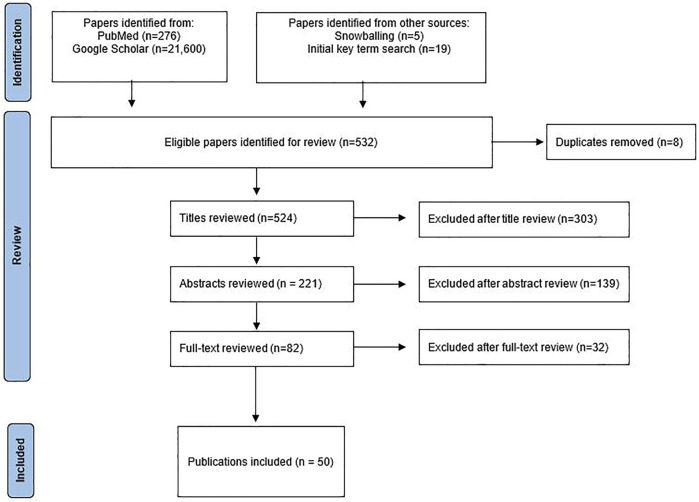
Publication search and selection process.

Figure 1 shows the PRISMA flow diagram of the paper inclusion and exclusion process. The review yielded 50 eligible publications for full-text analysis; 36 were either program reports or studies, of which 14 were from the United States. The rest were carried out in West European countries, as well as one study from Israel and one from New Zealand. Table 1 presents a précis of the selected publications.

**TABLE 1:** Included papers by author, year, type and country.

Authors	Year	Type	Country
Bailey et al.	2016	Qualitative interview	United Kingdom
Goetzmann et al.	2009	Cross-sectional survey	Switzerland
Tong et al.	2012	Qualitative interview	New Zealand
Fortin et al.	2008	Qualitative study	Canada
Ross	2010	Pilot study proposal	United States
Mamode et al.	2013	Systematic review	United Kingdom
Kranenburg et al.	2007	Mixed method study	Netherlands
Kranenburg et al.	2004	Discussion paper	Netherlands
Maple et al.	2014	Cross-sectional survey	United Kingdom
Lima et al.	2012	Program report	Portugal
De Klerk	2010	Program report	Netherlands
Jacobs	2004	Program report	United States
Slaats et al.	2018	Retrospective observational study	Sweden and Netherlands
Wadström et al.	2019	Longitudinal study	Sweden
Woodle et al.	2010	Program report	United States
Ross et al.	1997	Editorial	United States
Lennerling et al.	2007	Case studies	Sweden
Ghent et al.	2019	Interview study	Canada
Hanto	2007	Discussion paper	Canada
Azuri et al.	2013	Mixed methods study	Israel
Lewino et al.	1996	Exploratory descriptive study	United States
Dobbels et al.	2009	Cross-sectional survey	Belgium
Henderson et al.	2003	Cross-sectional survey	Canada
Annema et al.	2015	Cross-sectional survey	Netherlands
Albert	1998	Program report	United States
Ono et al.	2008	Cross-sectional survey	Brazil
Pronk et al.	2017	Longitudinal study	Netherlands
Dor et al.	2011	Terminology assessment	Netherlands
Adams et al.	2002	Conference report	United States
Morrissey et al.	2005	Program report	United States
Patel et al.	2011	Discussion paper	United Kingdom
Clayville	1999	Qualitative interview	United States
Mark et al.	2006	Program report	United States
Corr et al.	1994	Discussion paper	United States
Matas et al.	2000	Program report	United States
Gohh et al.	2001	Case study discussion	United States
Colaneri	2004	Discussion paper	United States
Erim et al.	2010	Program report	Germany
Jendrisak et al.	2006	Program report	United States
Thiel et al.	2001	Discussion paper	Switzerland
Gilbert et al.	2005	Program report	United States
Wallis et al.	2011	Program report	United States
Durand et al.	2014	Qualitative interview	Canada
Olbrisch	2001	Discussion paper	United States
Hilhorst	2005	Discussion paper	Netherlands
Rodrigue et al.	2011	Cross-sectional survey	United States
Landry	2006	Discussion paper	United States
Lucan	2007	Program report	Romania
Duvoux	2019	Program report	Canada

Table 2 shows the reason types coded for and against anonymity. The first column shows the broad reason types, the “Side” column represents whether the narrow reasons were for (“pro”) or against (“con”) anonymity. The third column contains the narrow reasons, those that we did not find pertinent to paired donations are marked with an asterisk (*). The fourth column shows the donation context in which the narrow reasons were found: unspecified, deceased donor (Deceased) or KPD (Paired). Reasons showing more than one donation type indicate that they were found in more than one circumstance. For instance, while the reason “anonymity is the standard that protects donors and recipients” was only found in papers that reported unspecified donations, reason “shields burden of knowledge in case of negative outcomes in the other party” was found stated in all three donation types.

**TABLE 2:** Reasons for and against anonymity.

Broad reasons	Side	Narrow reasons	Donation type	Source
Protection against harm	Pro	Feelings of guilt from meeting the donor family can affect organ integration^a^	Deceased	(6, 7)
		Anonymity is the standard that protects donors and recipients	Unspecified	(8, 9)
		Shields burden of knowledge in case of negative outcomes in the other party	Paired, unspecified, deceased	(9–16)
		Anonymity can protect donors from being pressured or coerced to donate	Paired, unspecified, deceased	(6, 14, 17–21)
		People are protected from feeling indebted, guilt and expected to say thanks	Paired, Unspecified, Deceased	(6, 8, 9, 18, 22–28)
		Contact with the other party can lead to great emotional stress	Paired, unspecified, deceased	(1, 12, 18, 22–25, 29, 30)
		Meeting may lead to unequal relationships between parties^a^	Unspecified	(31)
		Awkwardness and discontent when anonymity was breached by the other party	Unspecified	(17, 31)
		Meeting the donor family can lead to recipients feeling pressured to nurture the organ^a^	Deceased	(22)
		Prevents risks of financial extortion, abuse, blackmail, organ trafficking or expectations of secondary gains	Paired, unspecified, deceased	(18, 25–27, 32, 33)
		Bias or disappointed expectations of the other party	Paired, unspecified, deceased	(8–10, 17, 18, 22, 23, 28, 29, 31, 32, 34)
		Donor families became too involved in the recipient’s life	Deceased	(29)
	Con	Disadvantages were reported due to the lack of contact^a^	Deceased	(22)
		Signs of distress were found in parties in the absence of expressed thanks^a^	Deceased	(16, 17)
		Donors reported the experience to be lonely, business-like and impersonal^a^	Paired or unspecified	(18)
		There is no evidence of ulterior motives, exploitation or expectations of reciprocity to organ donation	Unspecified	(9, 35, 36)
General benefits	Pro	The benefit of meeting does not justify the workload involved in facilitating it	Unspecified	(23)
		Anonymity gives people closure, relief and opportunity to focus on their own lives	Paired, unspecified, deceased	(10, 22)
		Meetings have been positive and beneficial	Paired, unspecified, deceased	(2, 14, 17, 19, 22, 29, 37, 38)
		Good relationships were formed	Paired, unspecified, deceased	(9, 17, 22, 23, 30, 31, 39, 40)
	Con	Expressing gratitude in person can help establish a bond	Unspecified	(36)
		Receiving gratitude can have a healing effect for the donor family^a^	Deceased	(1, 22, 25, 29, 40, 41)
		Removing anonymity helps people achieve closure, better quality of life and promote wellbeing	Paired, unspecified, deceased	(17, 18, 40)
		Seeing the positive outcome in a recipient confirms the meaningfulness of the act	Paired, unspecified, deceased	(17, 22, 29–31, 38, 41, 42)
		Anonymity prevents donors from the satisfaction of seeing the benefits of their act^a^	Unspecified	(42–45)
		Helps maintain transparency on the quality and origin of the organ	Paired	(39, 46)
Gratitude	Pro	Recipients can say thanks anonymously	Unspecified	(8, 23)
		Recipients for alcoholic liver disease and those with higher education felt no need to say thanks	Deceased	(1)
	Con	The primary reason to contact is to personally say thanks	Unspecified, deceased	(1, 11, 24, 25, 29, 31)
		Anonymity prevents people from their natural desire to express gratitude	Unspecified, deceased	(18, 23, 41)
		The opportunity to give thanks through a meeting should not be wasted	Paired, unspecified, deceased	(24, 36, 38)
Curiosity	Pro	Initial curiosities about the other party dissipate with time	Unspecified	(11, 19)
	Con	Donors and recipients are naturally curious about each other	Paired, unspecified, deceased	(1, 6, 12, 18, 25, 30, 31)
		Direct contact satisfied donor families’ curiosity	Deceased	(22)
Unrealistic to implement	Con	Strict anonymity can be difficult or impossible in some circumstances	Paired, unspecified	(10, 32, 39, 46, 47)
		People would try and manage to find each other despite restrictions	Paired, unspecified	(8, 48)
		Inapplicable when pairs are genetically or already emotionally involved with each other	Paired	(46)
Fundamental rights	Con	Making decisions for oneself is a fundamental right	Paired, unspecified, deceased	(1, 18, 24, 25, 29, 30)
		Donors have a right to know to whom their organ was donated	Unspecified	(8, 18)
		People are capable of making the best decisions for themselves	Deceased	(22, 30)
		People are responsible for the consequences of their own decision	Unspecified	(18)
Respect people’s wishes	Pro	Donors and recipients wish to remain private to prevent problematic relationships	Paired, unspecified, deceased	(1, 8–10, 12, 18, 22, 23, 25, 26, 32, 41)
		Donors and recipients do not feel the need to contact the other party	Paired, unspecified, deceased	(1, 8, 11, 15, 19, 22, 24, 27)
		Donors and recipients agree with anonymity	Paired, unspecified, deceased	(1, 11, 15, 18, 19, 27, 31)
		Donors want to feel like the donation was made to their loved one	Paired	(11)
	Con	Donors and recipients want to meet each other	Paired, unspecified, deceased	(1, 19, 22, 24, 25, 29, 30, 36, 39, 49, 50)
		Some would agree to meet because it could be important to the other person	Unspecified	(31)
		Anonymity should be lifted if everyone agreed to meet	Paired, unspecified, deceased	(2, 18, 24, 25, 29, 47, 51)
		Donors and recipients want to share the experience with each other	Paired, unspecified, deceased	(29, 31)
Professional neutrality	Con	Medical professionals should remain neutral	Deceased	(29)
		Medical teams should respect and facilitate people’s wishes to meet	Deceased	(22, 24, 29, 30)
Timing is important	Con	Anonymity during the early stages of the operation is important, but can be reassessed afterwards if others agree to meet	Paired, unspecified, deceased	(2, 8, 21,37, 42, 47, 52)
		A professional is needed to facilitate and give counselling to both parties before the meeting	Deceased	(1, 24, 29, 40)
		There is a preference to meet within 1 year of the transplantation	Deceased	(22)
		Gradual preparations before the meeting is needed	Deceased	(22, 24, 25, 29, 40)
Information disclosure	Con	Donors worry that the recipient’s lifestyle or non-adherence may cause a negative outcome	Unspecified	(34)
		Fear of acquiring the donor’s bad traits or personality through the grafted organ, and would like to have these traits pre-disclosed	Deceased	(6)
Altruism	Pro	Donate anonymously is true altruism^a^	Paired, unspecified	(8, 18, 26, 27, 42)
Reciprocity	Pro	The reciprocity principle of organ donation can be achieved despite anonymity^a^	Deceased	(53)
Donation pool	Pro	Direct living donation may lead to a decrease in the organ donation pool^a^	Unspecified	(8)
	Con	People with positive experience about organ donation can become strong advocates pro new donors^a^	Unspecified, deceased	(1,9,17,24,25)
		Anonymity might discourage people who need a personal story from becoming donors^a^	Unspecified	(18)

^a^ Reasons that are not applicable for paired donations.

In total, we identified 62 narrow reasons and 13 broad reasons. There were 24 narrow reasons in favor of anonymity and 38 against. Further, the most frequently cited narrow reason for anonymity were those coded under the “protection against harm” broad reason type (*n* = 12). In reasons against anonymity, we identified eight narrow reasons that were coded “respect people’s wishes” broad reason type, four narrow reasons for “fundamental rights” and four narrow reasons for “timing is important” broad reason types.

### Protection Against Harm

We found that anonymity as a preventive measure against potential harm was a frequently given reason for those in favor of its legal imposition. This included safeguarding donors from “burden of knowledge in case of negative outcomes,” protecting recipients from “feeling indebted, guilt and expected to say thanks,” as well as preventing possible “awkwardness,” “emotional stress” and fears of bias overall.

Several papers reported negative donor-recipient interaction experiences, due to bias related to social and religious differences, or unmet expectations of the other person (18, 22, 29). Two studies reported cases of unintended donor-recipient meeting during hospitalization. In one study, two donors had intentionally breached anonymity without their recipient’s consent (17); the other study reported two accidental donor-recipient meetings (31). In both studies, one person reported discontent and regretted the meeting, while another was pleased despite the initial awkwardness.

Further, we found concerns regarding risk of financial extortion, blackmail or expectations of secondary gains from donors to recipients, or that potential donors may be coerced into donation if anonymity was not maintained. However, a study on motivations for unspecified donations found that donors commonly thought the act would “make a huge difference to someone else’s [life]” (15). Other studies showed no evidence of ulterior motives, expectations of reward from organ donors (9, 35, 36). We did not find any report of forced donations.

Some narrow reasons in favor of anonymity were less clear. For instance, Azuri et al. (22) argued that meeting the deceased donor family might lead the recipient to feel an “extra sense of responsibility to nurture the donated organ,” but we found no further clarifications on this reasoning.

### General Benefits and Gratitude

In the “general benefits” broad reason, we identified two narrow reason types for anonymity and eight against. One reason that supported anonymity was that it allowed donors or donor families and recipients to achieve their own closure and to focus on their own lives (10, 22). The other was that the benefits of the meeting do not justify the resource cost of facilitating them (23).

In narrow reasons against anonymity, we found observed benefits of the donor-recipient meeting: people were able to achieve closure together, good relationships were formed and meetings were generally reported as positive and beneficial. Further, donors reported that seeing the positive effects of the transplantation brought a sense of satisfaction and meaningfulness to their act. Two studies argued that lifting anonymity in KPDs can help maintain transparency on the quality and origin of the organ (39, 46).

In “Gratitude” broad reason, we identified five narrow reason types, two in favor of anonymity and three against. In narrow reasons against anonymity, one was cited in six papers, arguing the primary reason for people to wish contact was to personally say thanks. Other narrow reasons against anonymity argued that it prevents people’s natural desire to give thanks, and the opportunity to do so through a meeting should not be wasted. In reasons for anonymity, two papers stated that gratitude can be expressed anonymously. In addition, one paper found that liver recipients in particular felt no need to express gratitude.

### Curiosity

For broad reason type “Curiosity,” we identified three narrow reasons, one for anonymity and two against. Whereas donors and recipients stated curiosity being a reason for wanting to relinquish anonymity, two studies that presented an argument against suggested that these curiosities tend to dissipate with time (11, 19).

### Unrealistic to Implement

We identified three narrow reasons that argued strict anonymity would be unrealistic, nearly impossible to maintain under certain circumstances: when the transplantations take place in the same institution and carried out by the same team (46), or when donors and recipients had to be hospitalized on the same floor (32). Authors from two papers stated in countries where conditions allow, people would try and succeed in finding each other, despite anonymity restrictions (8, 48).

### Fundamental Rights, Respect People’s Wishes and Professional Neutrality

Under “Fundamental rights,” we identified four narrow reasons that were against anonymity, of which two that we found closely related to several narrow reasons under “respecting people’s wishes.” For instance, donors and recipients of Slaats et al. study stated that people should be free to make choices on their own anonymity, and be responsible for the consequences of such decision. Other papers argued that anonymity should be lifted if both parties agreed to do so (18, 24, 25, 29, 47, 51).

Further, two narrow reasons that were identified under the “professional neutrality” broad reason were presented under similar contexts to those coded under “respect of people’s wishes.” Deceased donation families and recipients expressed that medical professionals should remain neutral, respect and facilitate people’s wishes to meet (22, 24, 29, 30).

### Timing Is Important

For broad reason “Timing is important,” we identified four narrow reasons, all were against maintaining anonymity after the operation. We found an emphasis on the importance of maintaining pre-transplantation anonymity, but the donor and recipient’s decision to meet can be reassessed by the medical professionals afterwards (8, 37, 42, 47, 51, 52).

Further, findings from studies in deceased donations showed participants had a preferred delay period between time of surgery and time to meet. For instance, Azuri et al. (22) found two preferred post-surgery delays: within a month or at least 1 year after.

## Discussion

Overall, we found that the most frequently given reasons in favor of anonymity were concerns for potential harm that may arise from donor-recipient interactions, whereas reasons against anonymity were argued based on the observed benefits associated with the organ donor-recipient interactions. While frequency is not the prime objective of our study, it suggests nonetheless that potential harm being a common concern, despite the lack of empirical evidence, and that further research may be required. Other main findings from our study were concerns for respecting people’s “Fundamental rights.” Interestingly, these were often argued along with “Respect people’s wishes,” “Professional neutrality” and “Timing is important,” which suggests people perceiving them as being closely associated with the respect of people’s decisions as a fundamental right.

In terms of “Protection against harm,” we found few studies that reported “harm” observed from the donor-recipient interaction, including two reports of discomfort, awkwardness and regret having met the other party, when anonymity was breached without their consent.

For concerns of blackmailing, extortion or coercion, we did not find any evidence of ill-intents in our review. While our findings do not rule out their potential occurrence, it is unlikely to be frequent. First, most countries have signed the WHO Guiding Principles that condemn commercialization of organs (54), in addition to national legislations against monetary procurement of organs. Second, ill-intents and wrongdoings are arguably possible if meetings occur before the donation, not after it. Further, risks may be disclosed to KPD pairs before the operation, and preventive measures against concerns of harm can be implemented by the medical team afterwards.

In contrast to concerns for potential harm, we found reports of observed donor-recipient interaction benefits, including good relationships being formed; for donors, seeing the positive outcome in the recipient reportedly affirmed the meaningfulness of their act.

In unspecified and deceased donations, some authors argued that the donated organs were often seen as the “gift of life” (41)—which explains recipients who were reportedly keen to express gratitude for receiving the “gift.” In these donations, recipients reported strong, positive emotions that motivated them to do so personally. For KPDs, circumstances may differ, since anonymity reportedly allowed some donors to keep the procedure as though the organ was donated to their intended recipient (12). In this case, it would be justifiable to respect the donors’ wishes, but not as a reason to legally impose anonymity.

Indeed, legally mandated anonymity excludes donors from all possibility of seeing the positive impact of their act, or recipients to form a good relationship with their donor, especially if both pairs wish to make contact. These elements should be considered, since it is arguably human nature for donors to wish seeing the positive outcome of their act, upon explicit agreement from the recipients.

Another possible outcome to consider is the fear of bias. Participants from studies reported stress due to people seeing or fearing unmet expectations of their donor or recipient, including social or religious bias. The donation pairs should thus be informed of such risk, that the other pair may or may not possess their expected characteristics and *vice versa*, allowing people to decide whether they would make contact.

Given the scarce evidence of harm found in donor-recipient interactions, as well as the observed benefits amongst those who did meet, versus concerns for potential harms, findings of our study suggest that the strict anonymity policy should be reconsidered. This echoes the statement by Pronk et al. (2), that “discussion on the risks and benefits of anonymity in anonymous donation, has long been more speculative than evidence-based,” which we found equally applicable to the KPD context.

Therefore, we argue that revoking anonymity should be made possible, if all concerned persons made explicit and independent decision to do so, to “preserve the ethical principle and morality of autonomy” of the decision-making individual (30). This argument is in line with the *Directive 2010/53/EU,* which recommends the possibility of revoking anonymity after transplantation. In practice however, this is generally not allowed by the domestic laws of European members States.

On the other hand, the free-decision approach is already in practice in many countries, where post-operation anonymity may be relinquished if all parties agree, such as the United States, Switzerland and the United Kingdom. In addition, studies in the Netherlands, Sweden and the United Kingdom showed respecting one’s decision to revoke anonymity to be well received by organ donation pairs: participants expressed satisfaction in the decision to remain anonymous, and donation pairs who opted to meet generally reported positive experience from their interaction (2, 18).

In this light, the decision approach should maintain the requirement for all persons of the donation pairs to consent, in the objective of upholding the principle of respecting people’s decisions. If one person in the paired donation wishes to maintain his or her anonymity, then that wish should be fully respected and upheld for both donation pairs.

We found papers that went one step further and stressed the importance of professional neutrality, with respect to the donors and recipients’ wishes—that professional follow-up plays a key role in regulating and maximizing the safeguard of the couples’ wellbeing in carrying out their decisions.

What could this look like in practice? First, we found that medical professionals, donors and recipients in general agreed that anonymity should be upheld before the operation. Prior to the surgery, however, donation pairs may already be informed of their right to revoke their anonymity afterwards, if everyone gives their independent and explicit consent. The discussions between medical professionals and each individual is thus critical to allow informed decisions.

Second, during and after the transplantation process, counselling and advice as a preventive measure against potential harms. These sessions may inform donation pairs the possible risks and benefits of interaction, as well as the possibility of a negative outcome. The informed knowledge of unequal outcomes is already in practice in the United States, mandated by the Organ Procurement and Transplantation Network policy for KPDs (art. 13.4.c.11).

Third, ensuring sufficient time between the surgery and moment of decision on anonymity can allow people to reflect, discuss and seek further professional advice if needed. The time delay is likely to be important, since studies suggest that initial curiosities about the other party tend to dissipate with time (11, 19). A time delay would allow initial curiosities to wane, so those who are truly keen on making contact may benefit from its advantages.

Despite the positive effects reported from donor-recipient interactions, Ghent et al. (23) argued that successful meetings do not justify the resource cost of facilitating them, because they could be used on transplantation work instead. This brings to question how effectively the resources were allocated in staff time and other resources attributed to the task. Since revoking anonymity by consent is already in practice in multiple countries with reported positive outcomes, we argue that finding the appropriate resource needed may be worthwhile, so people may enjoy and share the benefits of the act.

In addition, we noted that publications on anonymity between donation pairs were relatively scarce in Europe and other countries, compared to the United States. This may due to cultural differences, as theorized by authors who noted differences in opinions on anonymity between study participants of different countries. Cultures with blurred personal boundaries may have stronger wish for solidarity over personal privacy (22). Further, whereas European cultures favor following a “collective norm,” American societies appreciate individual opportunities (1). This suggests that anonymity merits further investigations, so that each national policy caters to its domestic needs.

## Strengths and Limitations

To our knowledge, this is the first systematic review of reasons that investigated applicable ethical reasonings regarding anonymity for paired organ donations. Further, this paper highlights the disadvantages and advantages of maintaining or lifting paired organ donation anonymity from ethical practical perspectives.

Our review also has several limitations. First, coding text passages into narrow and broad reason types had a risk of bias. To minimise this, researchers worked independently, and text passages were reviewed based on their context during coding, to avoid interpretations outside the contextual scope of the paper from which it was extracted. However, as with all subjective interpretations, this method is not entirely free of reviewer bias. Second, we perceived a loss of detailed information during the coding process.

Third, despite the deliberate broadened search, nearly all of our findings were in English, with more eligible papers from the United States than any other country, which could have led to cultural bias in the findings of the eligible papers. This could be due to the search being conducted in English prominent databases. While this was addressed by placing no language restrictions, which generated two non-English articles, there may be other country or region-specific search engines that could have generated more results from non-anglophone countries with different cultural and legal views.

Fourth, while we broadened our search in the key terms used, we noticed certain papers that were pertinent to our review could only be found by applying the snowballing technique. Consequently, there may be papers that are pertinent to this review but did not show up in our search.

## Conclusion

In sum, while we found a wealth of reasons for and against anonymity in organ donations, those that supported anonymity were primarily based on speculation without supporting evidence. In contrast, we found reasons against anonymity that were based on observed benefits. Therefore, we did not find reasonings that justified legally imposed anonymity for donation pairs who wish to make post-operation contact. In fact, we found that the most ethically convincing reasons to be those that emphasized the respect of an adult person’s capacity and right to make informed decisions for oneself, with professional support, careful evaluations and appropriate delay between times of operation and contact. This was supported by positive outcomes reported from donor-recipient interactions, where such practice was allowed. We thus deem that future research will be useful, to investigate the best timing for donors and recipients to make informed decisions on their anonymity, as well as the best clinical and medical practice to help prepare donation pairs to meet, if they so choose.

We also noted that countries that enacted regulations to allow relinquishing anonymity by consent, such as Switzerland, the United Kingdom and the United States, show a recognition and intent to preserve an individual’s autonomy. In contrast, other countries, including European states, maintain strict anonymity with no possibility of revoking donor-recipient anonymity. In light of our findings and of ethical considerations for best practice, we encourage policymakers to reconsider strict anonymity regulations for paired donations, to help maximizing donors and recipients’ benefit from their organ transplants.
